# Academic Fatigue of Young Researchers: The Price of the Publish or Perish Culture

**DOI:** 10.7759/cureus.97223

**Published:** 2025-11-19

**Authors:** José Emiliano González Flores

**Affiliations:** 1 School of Medicine and Health Sciences, Tecnológico de Monterrey Campus Ciudad de Mexico, Mexico City, MEX

**Keywords:** academic fatigue, medical education, mental health, publish or perish, research culture

## Abstract

The publish or perish culture has become a defining feature of modern academia, where career advancement often depends on publication quantity rather than scientific depth. This environment exerts intense psychological and ethical pressure on early-career researchers, contributing to stress, burnout, and declining research quality. Studies have linked publication pressure to increased depressive symptoms, sleep disturbances, and even research misconduct, underscoring the systemic nature of the problem. This editorial discusses how such pressures threaten innovation and scientific integrity while proposing reforms in evaluation metrics, mentorship, and mental health support to restore balance and sustainability within academic research.

## Editorial

In contemporary science, professional success is often measured not by the depth or originality of ideas but by the number of publications. The phrase "publish or perish" captures the structural pressure that defines the academic world, especially for young researchers navigating an increasingly competitive and resource-limited environment [1]. For many, publishing has shifted from being a pursuit of discovery to a necessity for career survival, replacing creativity and exploration with constant pressure to meet productivity targets. This editorial calls for academic institutions to revalue quality, collaboration, and mental health as essential pillars of a research system that seeks truth and serves society.

Recent research on academic burnout during the COVID-19 era highlights how institutional resources and psychological support play a critical role in sustaining engagement and preventing exhaustion among students and early-career researchers [2]. Recent surveys report high rates of depressive symptoms, sleep disorders, and early attrition from research careers linked to these pressures [3]. Some universities and research institutions have begun implementing wellness programs, mentorship initiatives, and workload reforms to address these concerns, yet such efforts remain inconsistent and often underfunded. Greater institutional commitment to mental health and sustainable research practices is essential to prevent the erosion of motivation and creativity among young scientists.

Beyond mental health, the publish or perish model also distorts scientific ethics and quality. In the race to publish, researchers may be tempted to fragment data into multiple minimal papers (salami slicing) or prioritize predictable, low-risk projects [4]. Such practices stifle innovation and undermine the core mission of science: to produce reliable, meaningful, and reproducible knowledge.

Paradoxically, the academic community has created a system where publication quantity is mistaken for scientific merit and where indices such as the h-index or impact factor overshadow the true value of curiosity-driven inquiry [5]. This paradigm risks transforming research into a race for visibility rather than a quest for truth.

Reforming this culture requires rethinking how we assess academic success. Institutions should adopt metrics that emphasize quality, collaboration, and societal relevance over mere productivity counts. Mentorship programs, open discussions about mental health, and recognition of teaching, clinical, and outreach contributions as integral parts of scientific work are essential steps toward a healthier academic environment [2].

Knowledge thrives in spaces that allow time to think, question, and fail. Freeing young scientists from the constraints of publish or perish does not mean lowering scientific standards; it means restoring science to its most noble purpose: the honest pursuit of truth in service of humanity.
